# Insulin Resistance, Inflammation, and Obesity: Role of Monocyte Chemoattractant Protein-1 (or CCL2) in the Regulation of Metabolism

**DOI:** 10.1155/2010/326580

**Published:** 2010-09-23

**Authors:** Anna Rull, Jordi Camps, Carlos Alonso-Villaverde, Jorge Joven

**Affiliations:** ^1^Centre de Recerca Biomèdica, Hospital Universitari Sant Joan de Reus, Institut d'Investigació Sanitària Pere Virgili, Universitat Rovira i Virgili, c/ Sant Joan s/n, 43201 Reus, Spain; ^2^Servei de Medicina Interna, Hospital Son Llàtzer, 07198 Palma, Spain

## Abstract

To maintain homeostasis under diverse metabolic conditions, it is necessary to coordinate nutrient-sensing pathways with the immune response. This coordination requires a complex relationship between cells, hormones, and cytokines in which inflammatory and metabolic pathways are convergent at multiple levels. Recruitment of macrophages to metabolically compromised tissue is a primary event in which chemokines play a crucial role. However, chemokines may also transmit cell signals that generate multiple responses, most unrelated to chemotaxis, that are involved in different biological processes. We have reviewed the evidence showing that monocyte chemoattractant protein-1 (MCP-1 or CCL2) may have a systemic role in the regulation of metabolism that sometimes is not necessarily linked to the traffic of inflammatory cells to susceptible tissues. Main topics cover the relationship between MCP-1/CCL2, insulin resistance, inflammation, obesity, and related metabolic disturbances.

## 1. Introduction

Metabolic syndrome is currently one of the most serious threats to human health and chronic systemic inflammation caused by tissue malfunction or homeostatic imbalance is a characteristic feature.Maintenance of homeostasis under diverse metabolic disorders is mostly associated with obesity and requires the coordination of nutrient-sensing pathways with the immune response.

Monocyte chemoattractant protein-1 (MCP-1 or CCL2) is a representative of the CC chemokine group,and its main known function is related to guiding monocytes to leave the circulation and become tissue macrophages, the first step in the initiation of inflammation. However, chemokines transmit cell signals that generate multiple responses, most unrelated to chemotaxis, that are involved in different biological processes. It is also frequently assumed that, in contrast to hormones, chemokines influence cellular activities in an autocrine or paracrine fashion. However, confinement to the well-defined environments of these actions is unlikely, and chemokines may be relevant effectors in chronic systemic inflammation. Specifically, alteration of plasma CCL2 concentration in metabolic disease states, the presence of circulating chemokines reservoirs, the recent evidence of novel mechanisms of action and certain unexplained responses associated with metabolic disturbances suggest the possibility that CCL2 may play a systemic role in the regulation of metabolism.

## 2. Systemic Chronic Inflammation is Related to Metabolic Disturbances

**Figure 1:**
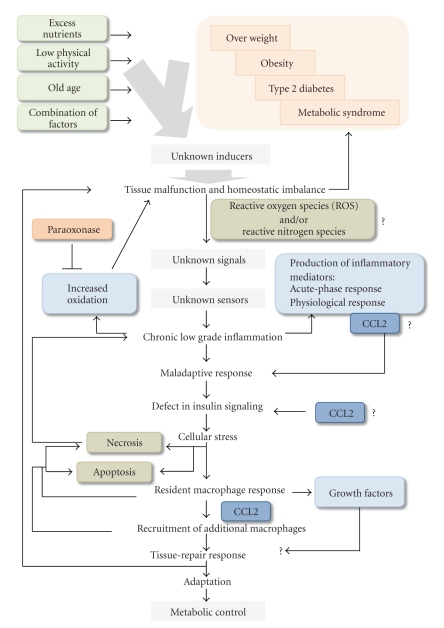
The inflammatory process and cellular metabolic control are convergent at multiple levels. Overnutrition, inactivity, old age, or a combination of factors triggers a systemic chronic inflammation associated with immune response which consists of a complex cellular adaptation in which chemokines play a crucial role.

The classical view of inflammation needs to be expanded to fully explain the inflammatory processes induced by adverse metabolic conditions and the accompanying deleterious effects in cells and tissues [1]. The sequence of events seems to be unaltered, and as such, the search for inducers and defining mediators remains a valid approach (Figure 1). The exact nature of the inducers that trigger the inflammatory response in tissues under metabolic stress is presently unknown, but these inducers are known to differ from those associated with infection and injury. Clinical experience suggests that such inducers are tightly associated with excess nutrients, a low level of physical activity, old age, or a combination of factors leading to overweight, obesity, type II diabetes, and/or metabolic syndrome. In all of these conditions, an excess of oxidation (mainly lipid oxidation) in cells, particularly in adipose tissue, is a common finding. This leads to the activation of inflammatory cells that further increase the oxidation in a vicious cycle that must be resolved [2–11]. Therefore, to date, candidate molecules for such inducers seem to be related to the production of reactive oxygen species (ROS) and/or reactive nitrogen species. ROS and the unstable balance between their production and the naturally occurring defenses against increased oxidation, by molecules such as paraoxonase, also have a role in converting lipoproteins into inflammatory signals by oxidizing their lipid and protein components [4, 5]. It is likely that accumulation of excess lipids may have biological effects that are signaled by unknown specific pathways. Regardless of the nature of such hypothetical inducers, they should be sufficient to trigger the production of inflammatory mediators, which in turn alter the normal functionality of many tissues and that can be classified into different groups according to their biochemical properties. Among such mediators, inflammatory cytokines (i.e., tumor necrosis factor-*α* (TNF-  *α*), IL-6) activate the endothelium and leukocytes and induce acute-phase responses. Particularly, chemokines (i.e., CCL2) control leukocyte extravasation and chemotaxis towards the affected tissues. However, the action of chemokines and other mediators cannot be limited to local effects; these molecules will likely display neuroendocrine and metabolic functions if we assume a more general role for inflammation in the control of tissue homeostasis [6].

Cells are normally in a basal state. A first and necessary step to induce a sequence of events is to stress these cells for which excessive availability of nutrients (a relatively modern alteration in humans) is a sufficient condition. The stress response consists of a complex, and not completely understood, cellular adaptation that is probably monitored by tissue-resident macrophages. Their basic functions include the removal of dead cells when necessary and maintenance of tissue homeostasis by a variety of tissue-specific mechanisms [7]. When cellular adaptation fails and malfunction becomes extreme, additional macrophages are recruited to help the tissues to adapt to these particular conditions of stress. The recruitment of macrophages in response to malfunctioning cells has been documented in adipocytes and hepatocytes, and increased production of CCL2 has been identified as a probable mediator [8, 9]. Obviously, if adaptation is no longer possible, the cells die. When macrophages recognize necrotic cells, a further inflammatory response will be induced, but alternatively, there may be a silent removal of dead cells if they are recognized as apoptotic. The consequence is that there is a net loss of cells; this likely needs to be compensated for by the generation of new cells of the same type. Such a process requires a subtle change in the role of macrophages and other cells to produce growth factors that promote cell proliferation in a tissue-repair response. The outcome is probably determined by additional unknown signals with intense effects on the overall metabolic control of the affected tissue and/or cells [10, 11].

## 3. How Does Obesity Initiate an Inflammatory Response? The Role of Macrophages, Endoplasmic Reticulum Stress, and Autophagy

There are a number of events associated with obesity that may result in the development of systemic inflammation, but how and when obesity might initiate an inflammatory response remains incompletely understood. It has been argued that large adipocytes completely consume the local oxygen supply, leading to hypoxia. This may activate cellular stress pathways, causing cell autonomous inflammation and the release of cytokines. Locally secreted chemokines attract macrophages into the adipose tissue located mainly around dead or dying adipocytes, forming characteristic crown-like structures. These macrophages release cytokines that further activate the inflammatory reaction in neighboring adipocytes, exacerbating local inflammation and expanding insulin resistance to other susceptible organs. 

**Figure 2:**
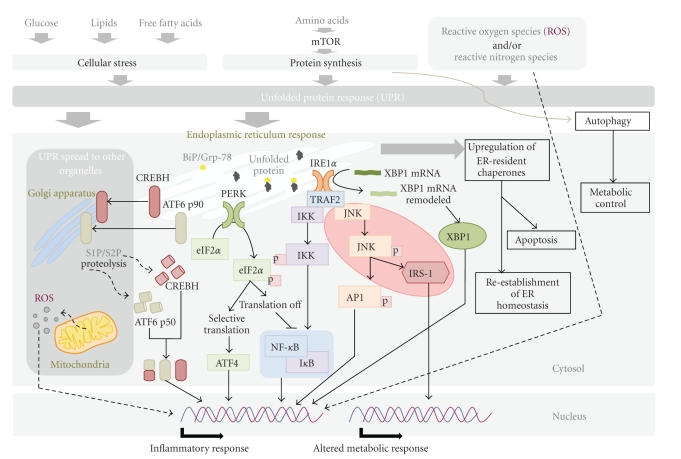
Inflammatory and metabolic responses may be interpreted as a response to cellular stress. Cells detect and react to stress in their environment. The endoplasmic reticulum is a principal contributor due to its central role in integrating multiple metabolic signals critical for cellular homeostasis.

The underlying mechanism inside cells probably depends on c-Jun N-terminal kinase (JNK) activation in insulin-sensitive tissues, that it is probably the principal mechanism by which the inflammatory signals interfere with insulin activity [12, 13]. The endoplasmic reticulum (ER) is a principal contributor to the various ways that cells sense stress because it plays a central role in integrating multiple metabolic signals critical for cellular homeostasis (Figure 2). In particular, the increased synthetic demand for energy availability challenges ER function, and alterations in cellular ER stress increase serine phosphorylation of IRS-1 in a JNK-dependent manner; a common finding in obesity, insulin action, and type 2 diabetes [14–16]. Regardless of the signals and sensors involved in this relationship, the role of the ER-protective response, known as the unfolded protein response (UPR), may be considered additive and complementary to the response of macrophages. Under mild conditions, the upregulation of chaperone proteins may re-establish ER homeostasis. If stimuli persist or the insult is intense, cell apoptosis is unavoidable [17]. The UPR is initiated by pancreatic ER kinase (PERK), inositol-requiring kinase (IRE1), and activating transcription factor 6 (ATF6) [18], three transmembrane proteins that mediate three different stress-sensing pathways; such pathways may lead to an inflammatory response. ER stress also elicits the production of ROS [19] (with consequent oxidative damage and activation of inflammatory signals), as well as the activation of the transcription factor cyclic-AMP-responsive-element-binding protein H (CREBH), which induces the production of acute-phase proteins [20]. Mitochondrial dysfunction may add further deleterious effects.

The sequence of events leading to the link between the UPR and the inflammatory response remains to be determined. UPR signaling is extremely sensitive to nutrients and plays a central role in the maintenance of glucose homeostasis and in the regulation of energy fluctuations in cells [21]. Moreover, recent findings demonstrated a central role for lipid chaperones (fatty acid-binding proteins) in the regulation of ER homeostasis in macrophages, and the ER responses can be modified to protect the organism against the deleterious effects of hyperlipidemia [22]. The ER stress responses are also linked to the mTOR pathway, which is essential for the regulation of numerous processes, including the cell cycle, energy metabolism, the immune response, and autophagy [23]. Recent findings have identified a critical function for autophagy in lipid metabolism that could have important implications for human diseases with lipid overaccumulation [24]. Although further research is necessary to firmly establish this paradigm, the regulatory and functional similarities between autophagy and lipolysis, along with the capability of lysosomes to degrade lipids, suggest that autophagy may contribute to breakdown of both lipid droplets and triglycerides [24, 25]. Unexpectedly, the effects of a loss of autophagy on hepatocytes differ from those reported for adipose tissue. In this tissue, autophagy functions to regulate body lipid accumulation by controlling adipocyte differentiation and determining the balance between white and brown fat [25]. In the liver (or other nonadipose organs) autophagy is protective preventing lipotoxicity via decreased hepatic lipid accumulation and promoting safer storage in adipose tissue.

## 4. The Contribution of Other Immune Cells to the Complications of Obesity

**Figure 3:**
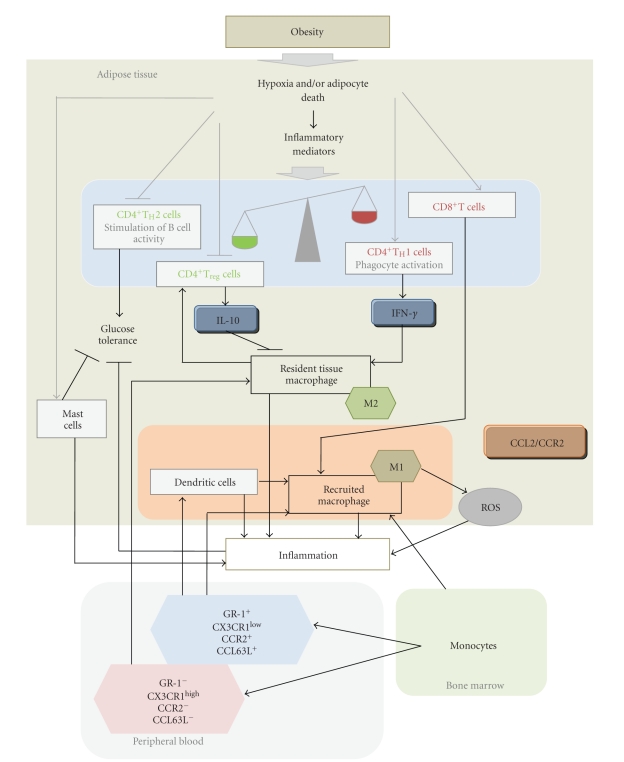
In obesity, immune cells other than macrophages may play a crucial role. To understand the heterogeneous inflammatory properties of adipose tissue macrophages, it is necessary to study the contribution of other immune cells to specific cellular response to metabolic stress.

Macrophages recruited to adipose tissue in subjects receiving a high-fat diet have unique inflammatory properties that are not observed in resident tissue macrophages [26]. Comparative analysis of gene expression between those recruited macrophages and the resident macrophages identified a total of 46 unique genes differentially expressed between the two populations. CCR2, which is required for recruitment of inflammatory macrophages, and genes important for macrophage activation, cellular adhesion, and migration are overexpressed in recruited macrophages. In lean mice, resident macrophages have low inflammatory activity; with obesity, newly recruited macrophages secrete pro-inflammatory cytokines. Although largely defined *in vitro*, it is generally accepted that macrophages can be classified in two different states: M1 and M2 [27, 28]. M1, or “classically activated” macrophages, are induced by proinflammatory mediators, show enhanced pro-inflammatory cytokine production, and generate ROS. At least in mice, diet-induced obesity leads to a shift in the activation state of macrophages from an M2-polarised state in lean animals (which may protect adipocytes from inflammation) to an M1 pro-inflammatory state (which contributes to insulin resistance) [29]. This obesity-induced switch of activation state seems to be coupled to the recruitment of a characteristic inflammatory subtype cells from the circulation [30], similar to what has been previously described for atherosclerotic lesions [31]. At least two major conclusions can be drawn from the above evidence. First, an intact CCL2/CCR2 axis, the principal chemotactic pathway, is necessary for understanding the mechanistic links between adipose tissue inflammation and the effects of obesity. Second, T cells may play a significant role as the plausible source of signals to initiate T helper-1 (T_H_1) responses through phagocyte activation, or humoral T_H_2 responses through stimulation of B cell activity. A recent array of studies substantially clarifies this issue [32–35]. Results from Nishimura et al. [32] support the notion that CD8^+^ T cells have an essential role in the initiation and propagation of adipose tissue inflammation in obesity. It was shown in diet-induced obesity that CD8^+^ T cells infiltrate into the epididymal fat pads before macrophage infiltration. Additionally, treatment with CD8^+^-specific antibodies, resulting in CD8^+^ T cell depletion, reduced M1 macrophage infiltration and ameliorated systemic insulin resistance in *ob/ob* mice. It can then be hypothesized that obese adipose tissue activates CD8^+^ T cells, which in turn recruit and activate macrophages. Winer et al. [33] performed a study based on the fact that some obese individuals progress to metabolic syndrome but others only have mild metabolic abnormalities [36, 37] and found that the progression of obesity-associated metabolic abnormalities is under the pathophysiological control of CD4^+^ T cells. Reconstitution of CD4^+^T cells, but not CD8^+^T cells, in lymphocyte-free obese Rag1-null mice improved glucose tolerance, enhanced insulin sensitivity, and lessened weight gain. Winer et al. [33] and Feuerer et al. [34] explored the ability of regulatory T cells (T_reg_) in adipose tissue to provide anti-inflammatory signals that block adipose tissue inflammation. T_reg_ cells normally account for 5%–20% of the CD4^+^ compartment but are thought to be one of the body's most crucial defenses against inappropriate immune responses [38, 39]. Visceral and subcutaneous adipose tissues have similarly low fractions of T_reg_ cells at birth, with a progressive accumulation over time in the visceral, but not subcutaneous, tissue [34]. This difference may be important given the association of visceral, but not subcutaneous, fat with insulin resistance [40, 41]. Visceral fat-derived T_reg_ cells overexpress a large number of genes that are not expressed in cells from the spleen, lymph nodes, and subcutaneous adipose tissue; these genes are mostly involved in leukocyte migration (e.g., CCR2) [34]. Extremely high levels of IL-10 transcripts were found that may block the production of inflammatory mediators. When most of the T_reg_ cells were ablated, pro-inflammatory transcripts (e.g., RANTES and CCL2) were strongly induced in the fat tissue, suggesting that the anti-inflammatory properties of T_reg_ cells may have therapeutic potential to inhibit elements of the metabolic syndrome [34]. In conclusion [32–34], obesity seems to alter the balance between T_H_1 and T_H_2 stimuli in fat, probably through depletion of T_H_2 cells and adipose tissue T_reg_ cells, increase in CD8^+^ and T_H_1 cells, or a combination of both effects, leading to the infiltration of macrophages that promote inflammation. At the same time, resident macrophages may communicate with adipose tissue T_reg_ cells to maintain homeostasis (Figure 3), and other inflammatory cells may be also contributors. For instance, mast cells are increased in the adipose tissue from obese subjects as compared to that from lean donors [35]. Furthermore, in mice receiving a high-fat, high-cholesterol diet, genetically induced deficiency of mast cells or their pharmacological stabilization (via disodium cromoglycate or ketotifen) reduces body weight gain and concentrations of inflammatory cytokines and chemokines in serum and in adipose tissue [35]. The crucial role of CCL2 in the migration of immune cells remains to be determined, but it should be highlighted that recruited macrophages originate from monocytes produced in the bone marrow. These monocytes give rise to two subsets of peripheral blood monocytes. One subset (GR-1^−^, CX3CR1^high^, CCR2^−^, and CCL62L^−^ monocytes) produces resident tissue macrophages, and the second subset (GR-1^+^, CX3CR1^low^, CCR2^+^, and CD62L^+^ monocytes) is preferentially recruited to inflamed tissues and gives rise to macrophages and dendritic cells [42].

## 5. The Role of CCL2 Regulating Inflammation and Metabolic Disorders

The crucial question of what initiates the activation and infiltration of relevant cells in adipose tissue and whether this constitutes an absolute requirement remains unanswered. Hypoxia, adipocyte death, or both [43, 44] (as a response to a metabolic overload) may be responsible for the fat infiltration of inflammatory cells but secretion of chemokines, mainly CCL2, is a necessary condition. 

The absence of CCL2 or CCR2 in LDLR^−/−^ and ApoE^−/−^ backgrounds protects these mice from developing atherosclerotic lesions, a condition in which macrophage recruitment and lipid overload play a crucial role. In these and other more complicated models, the CCL2/CCR2 axis may represent a common pathway for many proatherogenic factors [45–49] and plays a central role in monocyte recruitment, lesion formation, and vascular repair. However, data may vary under different experimental conditions and seem to be dependent on the metabolic status of the mice. In particular, the putative role of CCL2 appears to differ between normo- and hyperlipidemic models. The interpretation of data in these models is difficult because the expression of other chemokine genes, with redundant actions, is highly influenced by both the absence of CCL2 and the presence of dietary fat and cholesterol [50].

### 5.1. CCL2 Tissue Expression and Its General Impact in Metabolism

**Figure 4:**
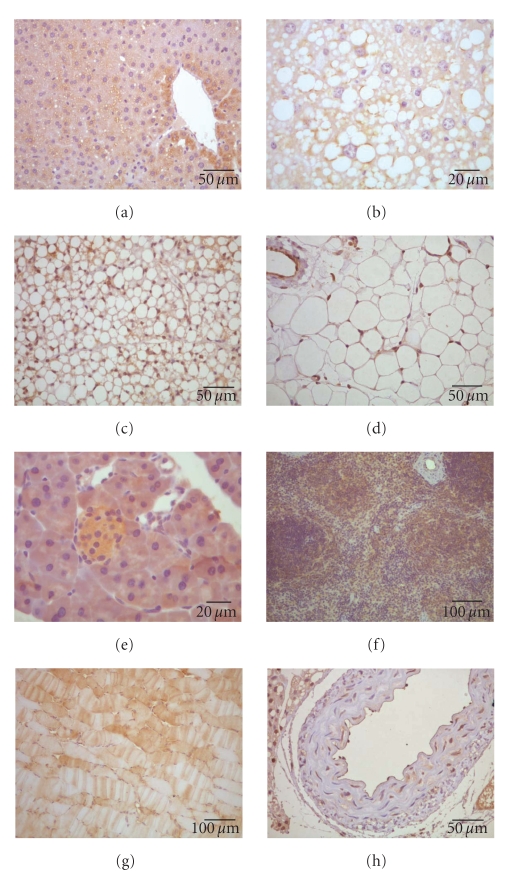
CCL2 is ubiquitously expressed and may be found in multiple cell types. It is easily detected via immunohistochemistry in tissues related to metabolism, including the liver (a), where it is also located in the periphery of lipid droplets (b), brown (c) and white (d) adipose tissues, pancreas (e), spleen (f), muscle (g) and aorta (h).

CCL2 is produced either constitutively or after selective induction (via oxidative stress, cytokines, or growth factors) by many cell types, including fibroblasts as well as endothelial, epithelial, smooth muscle, mesangial, astrocytic, monocytic, and microglial cells. It is also found in hepatocytes, adipocytes, and islet cells, and some authors consider that it is present in virtually every tissue [8, 9, 51–54] (Figure 4). Such ubiquity suggests an endocrine rather than paracrine function, as well as an important function in several biological processes. Thus, CCL2 has been implicated as a potential target in many disease states [55], including liver diseases [56] and insulin-resistant states [57]. However, it should be noted that knockout mice for CCL2 and its receptor are viable, although with minor defects, [58]; thus CCL2 may have effective surrogates. It is plausible that in the absence of CCL2, other chemokines may function effectively, but the data suggest important and pleiotropic functions. In particular, CCL2 may contribute to pathologies associated with hyperinsulinemia [57], given that *ccl2* is an insulin-responsive gene that may alter adipocyte function. Both the adipose tissue expression and circulating concentrations of CCL2 increase in obesity and decrease following treatment with thiazolidinediones [58, 59]. In a mouse model of diet-induced obesity, CCR2 deficiency attenuated the development of obesity, adipose tissue macrophage accumulation, adipose tissue inflammation, and systemic insulin resistance. Also, in mice with pre-existing obesity, short-term pharmacologic antagonism of CCR2 reduces adipose tissue macrophage content and improves *in vivo* insulin sensitivity [60]. However, the absence of CCR2 has no measurable metabolic effect in lean animals. Subsequent studies in CCL2-deficient mice suggest that CCL2 plays a minimal role in glucose metabolism and insulin sensitivity in mice fed a normal diet, but is important for pathogenic macrophage infiltration into adipose tissue, insulin resistance, and hepatic steatosis induced by a high-fat diet [61]. Moreover, studies in transgenic mice that over-express *ccl2* under the control of the adipose tissue-specific AP2 promoter indicate that CCL2 in adipose tissue, *per se*, induces macrophage recruitment and insulin resistance [62, 63].

### 5.2. CCL2/CCR2 Pathway and Insulin Resistance in Obesity

The CCL2/CCR2 axis is a major component of insulin resistance in obese mice. Lipid peroxidation and the consequent oxidative stress and oversecretion of CCL2 have been recently implicated in early stages of adipose tissue inflammation [64–66]. Lysophosphatidylcholine (LPC) is a prominent component of oxidized low-density lipoproteins (LDL). During oxidation, 40% of LDL phosphatidylcholine can be converted to LPC by LDL-associated phospholipase A2 [64]. LPC stimulates the production of CCL2 by cells at the transcription level through a mechanism that involves MEK/ERK, tyrosine kinase, and (to a lesser extent) protein kinase C (PKC) activities [65]. More recent data suggest that 12/15-lipoxygenase (12/15 LO) is required for the early onset of high fat diet-induced adipose tissue inflammation and insulin resistance in mice [66]. Cells overexpressing 12/15LO secreted higher amounts of CCL2. Accordingly, adipose tissue from 12/15LO KO mice fed a high-fat diet was not infiltrated by macrophages, did not show any increase in inflammatory markers, and did not exhibit changes in the insulin-stimulated glucose disposal rate or hepatic glucose output.

### 5.3. CCL2 and Obesity-Associated Macrophage Recruitment Are Not Clearly Associated: Independent Effects on Metabolism

A note of caution has been recently introduced by Inouye et al. [67], who reported that the absence of CCL2 does not attenuate obesity-associated macrophage recruitment and appears to cause metabolic derangements, even in mice fed low-fat diets. Although the lack of macrophage recruitment may be masked by different experimental conditions, these results clearly indicate that CCL2 may have independent effects on metabolism that should be ascertained in future studies. Moreover, we recently described that CCL2-deficient mice, when rendered hyperlipemic by the concomitant ablation of the LDL receptor, demonstrate decreased lipoprotein clearance, derangements in free fatty acid delivery, and less glucose tolerance when fed regular chow [68]. These mice also show a partial resistance to alterations in glucose and lipid metabolism induced by dietary fat and cholesterol. LDLr^−/−^ and CCL2^−/−^LDLr^−/−^ mice have identical apparent phenotypes and similar body weight at 11-12 weeks of age. Both strains are hyperlipemic, but the CCL2^−/−^LDLr^−/−^ mice show higher plasma cholesterol and triglycerides, indicating a possible role for CCL2 in lipid metabolism. Further, we found similar but lower plasma cholesterol and triglyceride concentrations in CCL2^−/−^ mice as compared to wild type mice. Also, we found that CCL2^−/−^LDLr^−/−^mice show decreased *in vivo* [3H] VLDL catabolism as compared to LDLr^−/−^ mice. Interestingly, double KO mice also show a significant increase in plasma FFA concentration that is not observed in mice with only CCL2 deficiency. It is already documented that high plasma FFA concentration may cause peripheral insulin resistance, and that insulin resistance may also elicit decreased uptake of fatty acids by adipose tissue, promoting increased levels of circulating plasma FFAs [68] in a poorly investigated cycle. We therefore predicted a link between lipoprotein derangements and glucose metabolism that was confirmed with the observation of higher fasting plasma glucose concentration in CCL2^−/−^LDLr^−/−^ mice than in LDLr^−/−^ mice as well as a less intense and slower response to glucose overload in the double knockout mice. Taken together, these results suggest that hyperlipidemia, which is common in obesity and metabolic syndrome, may be a confounding factor, and that the absence of CCL2 may be as metabolically deleterious as overexpression of CCL2 in certain conditions. Under these circumstances, it is therefore possible that CCL2 may act as hormone rather than as a cytokine, although it remains to be ascertained whether CCL2 and LDLr share a common metabolic pathway.

### 5.4. CCL2 Mediates Biological Effects Other Than Leukocyte Chemotaxis

Chemotaxis is not the only known function for CCL2 [69]. For instance, CCL2-mediated angiogenesis has been demonstrated *in vivo* and appears to be independent of its induction of leucocyte recruitment [70]. Elevated CCL2 levels induce highly elevated expression of ER stress chaperones (mainly GRP78) that may protect against cell death. This has been established in postinfarct remodeling studies in transgenic mice with cardiomyocyte-targeted expression of CCL2 [71, 72], although apparently contradictory data have been found in CCL2-deficient mice [73]. Other evidence suggests that CCL2 is also involved in the cell expression of metalloproteinases, in the recruitment of cells active in the fibrotic process, and in protection against accumulation of oxidative stress proteins [69]. Moreover, signaling initiated by CCL2 binding to CCR2 triggers the induction of a novel zinc finger protein transcription factor that can induce cell death [74]. This factor, which has been called MCP-1-induced protein (MCPIP), causes the production of reactive oxygen and nitrogen species via the induction of NADPH oxidase and inducible NO synthase [75]. This oxidative stress causes ER stress that leads to autophagy and cell death. Interestingly, the interaction between CCL2, survival, and autophagy in the complex program of tumor progression has been previously suggested [76]. Whether other processes induced by CCL2 are also mediated via MCPIP remains to be ascertained.

### 5.5. CCR2 Is Not the Only Receptor for CCL2: Influence of Genetic Variation in Blood Concentrations of CCL2

Although CCR2 is the known receptor for CCL2 in tissues [77], another molecule, the Duffy antigen receptor for chemokines (DARCs) mediates the interactions of CCL2, with erythrocytes and endothelial cells [78]. Because DARC lacks completely the Asp-Arg-Tyr consensus motif in its second cytoplasmic loop, it cannot couple to G proteins and subsequent signaling pathways. Consequently, it has been grouped with two other heptahelical molecules, D6 and CCX-CKR, to form a family of atypical silent chemokine receptors [79]. However, it has been recently demonstrated that DARC does not act as a decoy but instead supports chemokine activity and is required for optimal chemokine-induced leukocyte migration *in vitro* and *in vivo* [80]. 

Several single nucleotide polymorphisms (SNPs) in the CCL2 gene have been reported to be related to blood concentrations of CCL2, but only rs1024611 (−2518 A/G) has been clinically replicated [81]. To identify the genetic basis of circulating CCL2 concentrations, a recent genome-wide association analysis has been conducted in three independent cohorts and the strongest association was for serum CCL2 with a nonsynonymous polymorphism, rs12075 (Asp42Gly) in *DARC*, indicating a possible role of vascular reservoir of pro-inflammatory cytokines. This association was supported by family-based genetic linkage at a locus encompassing the *DARC *gene underscoring the relevance of CCL2 pathophysiology for a broad spectrum of diseases [82].

### 5.6. CCL2 as a Therapeutic Target

A recent report has examined the effects of an increase in the plasma concentration of CCL2 resulting from short-term (acute) or long-term (chronic) administration of recombinant CCL2 in mice [83]. They found that a chronic increase in the circulating level of CCL2 induced insulin resistance, macrophage infiltration into adipose tissue, and an increase in hepatic triacylglycerol content, but an acute increase in the circulating CCL2 concentration also induced insulin resistance without macrophage infiltration into adipose tissue. In addition, the administration of a novel CCR2 antagonist ameliorated insulin resistance in mice fed a high-fat diet without affecting macrophage infiltration into adipose tissue. Taken together, their results indicate that an increase in the concentration of CCL2 in the circulation is sufficient to induce systemic insulin resistance irrespective of adipose tissue inflammation and suggest that CCL2 may be a direct effector in regulating metabolism. It is therefore conceivable that new therapeutic opportunities may arise from blocking of chemokine/receptor interactions with specific antagonists or blocking antibodies. Animal models have demonstrated effective reduction of lesion formation in coronary arteries and experimental in-stent restenosis [84, 85]. Surprisingly, such blockers have not been tested as antiobesity agents or as modulators of metabolic derangements, despite published promising results [86]. Whether such agents can be used in humans remains questionable because the effect of CCL2 suppression, as mentioned above, may not be absolutely safe. However, it may be possibly safer to transitorily decrease the expression of CCL2 with plant-derived flavonoids or interfere with CCL2-CCR2 interactions using small molecules currently under investigation for safety and effectiveness [87, 88]. Current studies are also being performed to test the hypothesis that metabolic disturbances may be alleviated through the modulation of CCL2 expression.

## 6. Concluding Remarks and Future Perspectives

Metabolic syndrome involving obesity, insulin resistance, type 2 diabetes, liver steatosis, and cardiovascular diseases is a critically important health issue associated with over-nutrition, inactivity, old age, or a combination of factors. Growing evidence supports the presence of a systemic chronic inflammation associated with immune imbalance in all of these disorders, where chemokines play a crucial role. Chemokines act as inflammatory mediators that trigger the cell stress response in tissues and produce a general response that is not limited to local effects but instead may be associated with the generation of multiple responses. Therefore, crosstalk between cells, hormones, and chemokines is fundamental for maintaining metabolic homeostasis. Specifically, CCL2 is a multifunctional chemokine implicated as a potential target in many disease states. CCL2 was first identified by its ability to regulate monocytes, macrophages, and other inflammatory cells at sites of inflammation, but it has recently been shown to be a major component of insulin resistance in obese mice. Moreover, *ccl2* is an insulin-responsive gene that decreases insulin-stimulated glucose uptake and increases the expression of adipogenic genes. Indeed, available data show ubiquitous expression of CCL2 that in turn may suggest an endocrine function similar to the action of hormones, which may explain its importance in several biological processes and its role in inflammation.

Future studies will need to address the possibility of new therapeutic treatments that reduce inflammatory recruitment and modulate chronic inflammatory processes but also improve metabolic disturbances through the modulation of CCL2 expression. The possibility of therapeutically and transiently modulating CCL2 with safe-plant flavonoids could offer clinical benefit.
